# Inpatient experience of anorexia nervosa

**DOI:** 10.1186/2050-2974-2-S1-P5

**Published:** 2014-11-24

**Authors:** Rachael Bellair, Susan Patterson, Esben Strodl

**Affiliations:** 1 Metro North Mental Health, Royal Brisbane and Women's Hospital, Brisbane, Australia; 2 Queensland University of Technology, Brisbane, Australia

## 

Anorexia nervosa (AN) is an extremely serious mental illness, with a high mortality rate and many debilitating physical and psychological symptoms. While hospitalisation is sometimes required for patients with AN there remains no evidence base for "best practice' inpatient treatment. With patients' views recognised as critical to improving efficiency and outcomes, calls have been made for more qualitative research into inpatients' experiences. In light of this the current paper utilised thematic analysis to examine 16 semi-structured interviews with inpatients diagnosed with AN, at a specialised eating disorders hospital unit. The study found an overarching theme of relationship ambivalence in connection with sub-themes of patients' eating disorders, eating disorder co-patients, staff and treatment. Participants' goals in relationship to their eating disorder and engagement in treatment shaped and were shaped by interactions with other inpatients with AN and staff. Clinical implications for this study and future research directions are discussed.

